# Supplementary figures and images for: A genome-wide screen in ex vivo gallbladders identifies Listeria monocytogenes factors required for virulence in vivo
Source: PLoS Pathog. 2025 Mar 3;21(3):e1012491. doi: 10.1371/journal.ppat.1012491 (PMC11892859; doi:10.1371/journal.ppat.1012491)

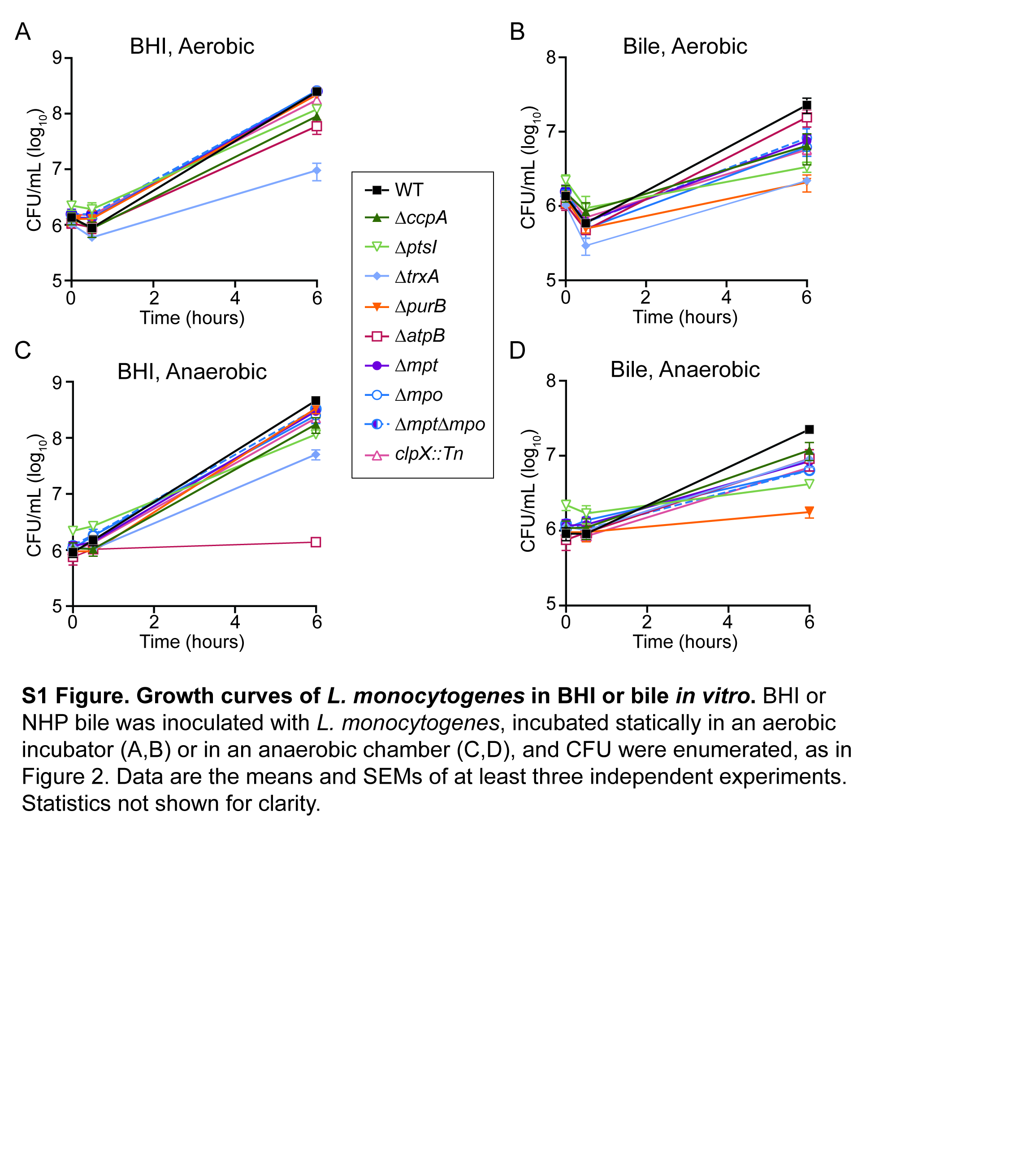

Supplement: S1 Fig — BHI or NHP bile was inoculated with L. monocytogenes, incubated statically in an aerobic incubator (A,B) or in an anaerobic chamber (C,D), and CFU were enumerated, as in Fig 2. Data are the means and SEMs of at least three independent experiments. Statistics omitted for clarity. (TIF) [file ppat.1012491.s001.tif]
