# Supplementary material for: A genome-wide screen in ex vivo gallbladders identifies Listeria monocytogenes factors required for virulence in vivo
Source: PLoS Pathog. 2025 Mar 3;21(3):e1012491. doi: 10.1371/journal.ppat.1012491 (PMC11892859; doi:10.1371/journal.ppat.1012491)
Supplement: S2 Table — (DOCX) [file ppat.1012491.s003.docx]

**S2 Table. Summary of significant Tn-seq hits. Highlighting indicates genes encoded in operons.**

| **Category** | **LMRG_** | **LMO** | **Name** | **Description** | **log2FC** |
| --- | --- | --- | --- | --- | --- |
| **Protein Homeostasis & Redox** | 00679 | 1233 | *trxA* | Thioredoxin | -2.27 |
|  | 01613 | 2219 | *prsA2* | Foldase | -1.75 |
|  | 02063 | 0964 | *yjbH* | Thioredoxin-fold protein | -1.58 |
|  | 00718 | 1268 | *clpX* | ATP-dependent Clp protease ATPase | -1.57 |
| **Nucleotide Transport & Metabolism** | 00558 | 1096 | *guaA* | GMP synthase | -2.28 |
|  | 02498 | 1773 | *purB* | Adenylosuccinate lyase | -2.16 |
|  | 01709 | 2538 | *upp* | Uracil phosphoribosyltransferase | -1.57 |
|  | 02485 | 0055 | *purA* | Adenylosuccinate synthetase | -1.53 |
| **Carbohydrate Transport & Metabolism** | 01396 | 1571 | *pfkA* | 6-phosphofructokinase | -2.52 |
|  | 02102 | 1002 | *ptsH* | PTS system, phosphocarrier protein HPr | -2.42 |
|  | 02103 | 1003 | *ptsI* | PTS system, enzyme I (EI) | -2.23 |
|  | 01691 | 2556 | *fbaA* | Fructose-bisphosphate aldolase class II | -2.24 |
|  | 02346 | 0097 | *mptC* | PTS system, mannose-specific IIC component | -1.66 |
|  | 02345 | 0096 | *mptA* | PTS system, mannose-specific IIAB component | -1.61 |
|  | 02347 | 0098 | *mptD* | PTS system, mannose-specific IID component | -1.61 |
|  | 00469 | 0781 | *mpoD* | PTS system, mannose-specific IID component | -1.65 |
|  | 00470 | 0782 | *mpoC* | PTS system, mannose-specific IIC component | -1.61 |
|  | 02869 | 0783 | *mpoB* | PTS system, mannose-specific IIB component | -1.60 |
| **Energy Production** | 01718 | 2530 | *atpG* | ATP synthase gamma chain | -1.98 |
|  | 01714 | 2534 | *atpE* | ATP synthase F0 sector subunit c | -1.91 |
|  | 01719 | 2529 | *atpD* | ATP synthase beta chain | -1.86 |
|  | 01720 | 2528 | *atpC* | ATP synthase epsilon chain | -1.85 |
|  | 01715 | 2533 | *atpF* | ATP synthase F0 sector subunit b | -1.84 |
|  | 01717 | 2531 | *atpA* | ATP synthase alpha chain | -1.78 |
|  | 01716 | 2532 | *atpH* | ATP synthase delta chain | -1.74 |
|  | 01713 | 2535 | *atpB* | ATP synthase F0 sector subunit a | -1.62 |
|  | 01386 | 1581 | *ackA* | Acetate kinase | -1.65 |
| **Regulation** | 02582 | 0289 | *yycH* | TCS YycFG regulatory protein | -2.72 |
|  | 01368 | 1599 | *ccpA* | Catabolite control protein A | -2.56 |
|  | 01447 | 1523 | *relA* | (p)ppGpp synthetase | -1.76 |
|  | 01787 | 2461 | *sigL* | RNA polymerase sigma-54 | -1.68 |
|  | 01038 | 1891 | *recU* | Holliday junction resolvase | -1.51 |
| **Coenzyme Metabolism** | 01048 | 1901 | *panC* | Pantoate-beta-alanine ligase | -2.91 |
|  | 01049 | 1902 | *panD* | 3-methyl-2-oxobutanoate hydroxymethyltransferase | -1.60 |
|  | 00813 | 1363 | *ispA* | Farnesyl diphosphate synthase | -1.58 |
| **DNA Recombination & Repair** | 01102 | 1955 | *xerD* | Site-specific tyrosine recombinase | -1.82 |
|  | 01101 | 1954 | *drm* | Phosphopentomutase | -1.47 |
|  | 01995 | 2702 | *recR* | Recombination protein | -1.66 |
|  | 01437 | 1533 | *ruvA* | Holliday junction ATP-dependent DNA helicase | -1.62 |
| **Amino Acid Metabolism** | 01304 | 1663 | *ansB* | Asparagine synthetase | -1.72 |
|  | 00749 | 1299 | *glnA* | Glutamine synthetase type I | -1.56 |
| **Lipid Metabolism** | 01083 | 1936 | *gpsA* | Glycerol-3-phosphate dehydrogenase | -1.71 |
| **Hypothetical** | 02932 | - | - | hypothetical protein | -1.92 |
