# Supplementary material for: A genome-wide screen in ex vivo gallbladders identifies Listeria monocytogenes factors required for virulence in vivo
Source: PLoS Pathog. 2025 Mar 3;21(3):e1012491. doi: 10.1371/journal.ppat.1012491 (PMC11892859; doi:10.1371/journal.ppat.1012491)
Supplement: S3 Table — (DOCX) [file ppat.1012491.s004.docx]

**S3 Table. Summary of mutant phenotypes**

|  | ***in vitro* phenotypes** | | | | ***in vivo* phenotypes (4 dpi)** | | | | |
| --- | --- | --- | --- | --- | --- | --- | --- | --- | --- |
| **Genotype** | **BHI^a^** | **Bile^a^** | **Plaque** | **BMDM^b^** | **GB** | **Liver** | **MLN** | **Spleen** | **Feces** |
| *∆ccpA* | +/- O_2_ | + O_2_ | ** | * | ns | ns | ns | ns | ns |
| *∆ptsI* | - O_2_ | +/- O_2_ | ns | * | *** | *** | *** | *** | ** |
| *∆trxA* | +/- O_2_ | +/- O_2_ | *** | * | *** | *** | ns | ns | *** |
| *∆purB* | ns | +/- O_2_ | *** | * | *** | *** | *** | *** | ns |
| *∆atpB* | +/- O_2_ | - O_2_ | ND | ns | ns | * | ** | ns | ** |
| *∆mpt* | ns | - O_2_ | ns | ns | *** | *** | ns | ** | * |
| *∆mpo* | ns | +/- O_2_ | ns | ns | * | ** | ns | ns | * |
| *∆mpt∆mpo* | ns | - O_2_ | ns | ns | * | *** | ns | ns | *** |
| *clpX::Tn* | ns | +/- O_2_ | *** | ns | *** | ** | ns | *** | * |

a. + or – O_2_ indicates the condition in which growth was significantly impaired compared to WT

b. Intracellular growth in BMDMs at 8 hours

ns = not significant

ND = not detected
