# Supplementary material for: A genome-wide screen in ex vivo gallbladders identifies Listeria monocytogenes factors required for virulence in vivo
Source: PLoS Pathog. 2025 Mar 3;21(3):e1012491. doi: 10.1371/journal.ppat.1012491 (PMC11892859; doi:10.1371/journal.ppat.1012491)
Supplement: S4 Table — (DOCX) [file ppat.1012491.s005.docx]

**S4 Table. Strains used in this study**

| ***E. coli* strains** | | |
| --- | --- | --- |
| **Strain** | **Description** | **Reference or Source** |
| XL1 | for strain construction | Stratagene |
| SM10 | for trans-conjugation | [1] |
| MLR-E893 | pLIM1 | Gift from A. Rietsch |
| MLR-E1156 | pLIM.ccpA-KO | This study |
| MLR-E1157 | pLIM.ptsI-KO | This study |
| MLR-E1158 | pLIM.trxA-KO | This study |
| MLR-E1159 | pLIM.purB-KO | This study |
| MLR-E1160 | pLIM.atpB-KO | This study |
| MLR-E1161 | pLIM.mpt-KO | This study |
| MLR-E1162 | pLIM.mpo-KO | This study |
| MLR-E006 | pPL2 | [2] |
| MLR-E1171 | pPL2.ccpA | This study |
| MLR-E1172 | pPL2.ptsI | This study |
| MLR-E1173 | pPL2.trxA | This study |
| MLR-E1174 | pPL2.purB | This study |
| MLR-E1175 | pPL2.atpB | This study |
| MLR-E1178 | pPL2.clpX | This study |
| ***L. monocytogenes* strains** | | |
| **Strain** | **Description** | **Reference or Source** |
| MLR-L001 | WT 10403S | [3] |
| MLR-L1148 | *∆ccpA* | This study |
| MLR-L1149 | *∆ptsI* | This study |
| MLR-L1150 | *∆trxA* | This study |
| MLR-L1151 | *∆purB* | This study |
| MLR-L1152 | *∆atpB* | This study |
| MLR-L1153 | *∆mpt* | This study |
| MLR-L1154 | *∆mpo* | This study |
| MLR-L1155 | *∆mpt ∆mpo* | This study |
| DP-L5566 | *clpX::Tn* | [4] |
| MLR-L1163 | *∆ccpA + ccpA* | This study |
| MLR-L1164 | *∆ptsI + ptsI* | This study |
| MLR-L1165 | *∆trxA + trxA* | This study |
| MLR-L1166 | *∆purB + purB* | This study |
| MLR-L1167 | *∆atpB + atpB* | This study |
| MLR-L1170 | *clpX::Tn + clpX* | This study |

**SUPPORTING INFORMATION REFERENCES**

1. Simon R, Priefer U, Pühler A. A broad host range mobilization system for in vivo genetic engineering: transposon mutagenesis in Gram-negative bacteria. Nat Biotechnol. 1983; 784–791. doi:10.1038/nbt1183-784

2. Lauer P, Chow MYN, Loessner MJ, Portnoy DA, Calendar R. Construction, characterization, and use of two Listeria monocytogenes site-specific phage integration vectors. J Bacteriol. 2002;184: 4177–4186. doi:doi.org/10.1128/JB.184.15.4177-4186.2002

3. Bécavin C, Bouchier C, Lechat P, Archambaud C, Creno S, Gouin E, et al. Comparison of Widely Used Listeria monocytogenes Strains EGD, 10403S, and EGD-e Highlights Genomic Variations Underlying Differences in Pathogenicity. MBio. 2014;5: e00969-14. doi:10.1128/mBio.00969-14

4. Zemansky J, Kline BC, Woodward JJ, Leber JH, Marquis H, Portnoy DA. Development of a mariner-based transposon and identification of Listeria monocytogenes determinants, including the peptidyl-prolyl isomerase PrsA2, that contribute to its hemolytic phenotype. Journal of Bacteriology. 2009;191: 3950–3964. doi:10.1128/JB.00016-09
