# Supplementary material for: A genome-wide screen in ex vivo gallbladders identifies Listeria monocytogenes factors required for virulence in vivo
Source: PLoS Pathog. 2025 Mar 3;21(3):e1012491. doi: 10.1371/journal.ppat.1012491 (PMC11892859; doi:10.1371/journal.ppat.1012491)
Supplement: S5 Table — (DOCX) [file ppat.1012491.s006.docx]

**S5 Table. Primers used in Tn-seq library preparation**

| **Primer name** | **Sequence** |
| --- | --- |
| olj376 | GTGACTGGAGTTCAGACGTGTGCTCTTCCGATCTGGGGGGGGGGGGGGGG |
| pJZ_RND1 | GATAAATTTGAATACTAGTCTCGAGTGGGGTACG |
| pJZ_RND2 | AATGATACGGCGACCACCGAGACACCACTCTAGAGACCGGGGACTTATCAGCC |
| TdT_Index_1_ATCACG | CAAGCAGAAGACGGCATACGAGAT**CGTGAT**GTGACTGGAGTTCAGACGTGTGCTCTTCCGATCT |
| TdT_Index_2_CGATGT | CAAGCAGAAGACGGCATACGAGAT**ACATCG**GTGACTGGAGTTCAGACGTGTGCTCTTCCGATCT |
| TdT_Index_3_TTAGGC | CAAGCAGAAGACGGCATACGAGAT**GCCTAA**GTGACTGGAGTTCAGACGTGTGCTCTTCCGATCT |
| TdT_Index_4_TGACCA | CAAGCAGAAGACGGCATACGAGAT**TGGTCA**GTGACTGGAGTTCAGACGTGTGCTCTTCCGATCT |
| TdT_Index_5_TCTGAA | CAAGCAGAAGACGGCATACGAGAT**TTCAGA**GTGACTGGAGTTCAGACGTGTGCTCTTCCGATCT |
| TdT_Index_6_GAGTTC | CAAGCAGAAGACGGCATACGAGAT**GAACTC**GTGACTGGAGTTCAGACGTGTGCTCTTCCGATCT |
| TdT_Index_7_CGAGAT | CAAGCAGAAGACGGCATACGAGAT**ATCTCG**GTGACTGGAGTTCAGACGTGTGCTCTTCCGATCT |
| TdT_Index_8_TCTCGA | CAAGCAGAAGACGGCATACGAGAT**TCGAGA**GTGACTGGAGTTCAGACGTGTGCTCTTCCGATCT |
| pJZTnSq_SeqPrimer | CACCACTCTAGAGACCGGGGACTTATCAGCCAAC |

Bolded sequences indicate sample-specific barcodes
